# Citrate metabolism in lactic acid bacteria: is there a beneficial effect for *Oenococcus oeni* in wine?

**DOI:** 10.3389/fmicb.2023.1283220

**Published:** 2024-01-04

**Authors:** Camille Eicher, Joana Coulon, Marion Favier, Hervé Alexandre, Cristina Reguant, Cosette Grandvalet

**Affiliations:** ^1^UMR PAM, Université de Bourgogne Franche-Comté, Institut Agro, Université de Bourgogne, INRAE, Dijon, France; ^2^Biolaffort, Floirac, France; ^3^Universitat Rovira i Virgili, Grup de Biotecnologia Enològica, Departament de Bioquímica i Biotecnologia, Tarragona, Catalonia, Spain

**Keywords:** lactic acid bacteria, citrate, *Oenococcus oeni*, metabolic engineering, citrate locus, proton motive force

## Abstract

Lactic acid bacteria (LAB) are Gram positive bacteria frequently used in the food industry for fermentation, mainly transformation of carbohydrates into lactic acid. In addition, these bacteria also have the capacity to metabolize citrate, an organic acid commonly found in food products. Its fermentation leads to the production of 4-carbon compounds such as diacetyl, resulting in a buttery flavor desired in dairy products. Citrate metabolism is known to have several beneficial effects on LAB physiology. Nevertheless, a controversial effect of citrate has been described on the acid tolerance of the wine bacterium *Oenococcus oeni*. This observation raises questions about the effect of citrate on the capacity of *O. oeni* to conduct malolactic fermentation in highly acidic wines. This review aims to summarize the current understanding of citrate metabolism in LAB, with a focus on the wine bacterium *O. oeni*. Metabolism with the related enzymes is detailed, as are the involved genes organized in *cit* loci. The known systems of *cit* locus expression regulation are also described. Finally, the beneficial effects of citrate catabolism on LAB physiology are reported and the negative impact observed in *O. oeni* is discussed.

## 1 Introduction

Citrate is an organic acid commonly found in food products like fruits, vegetables or milk. This organic acid can be metabolized by lactic acid bacteria (LAB) into aromatic compounds including diacetyl which is responsible for the buttery aroma which is desirable in dairy products such as butter, acid cream and cottage cheese, etc. In addition, CO_2_ produced from this metabolism contributes to the formation of “eyes” in certain types of cheese. Nevertheless, not all LAB can consume this organic acid. Indeed, its consumption depends on the presence of genes encoding a citrate permease and a citrate lyase enabling the internalization of citrate in the cell and the catabolism of citrate into oxaloacetate, respectively (Hugenholtz, 1993; Bekal et al., 1998a; Drider et al., 2004). In some species, these genes are plasmid-encoded, which explains the instability of this metabolic trait in LAB (Drider et al., 2004).

Several previous studies have reported the beneficial effect of citrate on the growth of LAB (Hugenholtz, 1993; Lolkema et al., 1995; Ramos and Santos, 1996; Magni et al., 1999; Kang et al., 2013). Indeed, the internalization of citrate and the conversion of oxaloacetate into pyruvate, leading to the consumption of one scalar proton, create a transmembrane proton motive force (PMF) which triggers the production of ATP through the F_0_-F_1_ ATPase (Ramos et al., 1994; Lolkema et al., 1995; Marty-Tesset et al., 1996). This mechanism participates in intracellular pH (pHi) homeostasis and consequently plays a role in the resistance of LAB to acid stress. In addition, in heterofermentative bacteria, citrate can be co-metabolized with sugars to facilitate the reoxidation of cofactors generated by hexose fermentation (Ramos and Santos, 1996; Zaunmüller et al., 2006; Kang et al., 2013). This co-metabolism results in an increased growth rate and the achievement of a higher final biomass (Ramos and Santos, 1996; Zaunmüller et al., 2006).

*Oenococcus oeni* is a LAB which come into play during the second step in wine fermentation. It has the capacity to consume malic acid to produce a weaker acid, namely lactic acid, in a process which deacidifies wine. In addition, by consuming all nutrient sources, the development of this bacterium in wine results in microbial stability. *Oenococcus oeni* improves the organoleptic properties of wine thanks to secondary metabolisms such as citrate metabolism (Bartowsky, 2005). Citrate is naturally present in small quantities in grape must (0.13 to 0.90 g/L; Alexandre et al., 2008). Its metabolism in *O. oeni* can lead to the production of acetate and lactate but also 4-carbon compounds: diacetyl, acetoin and 2,3-butanediol. Diacetyl synthesis is only desirable in very small quantities in wine. Indeed, its production is responsible for the development of a “buttery” character in wines that is undesirable beyond a certain threshold (Bartowsky and Henschke, 2004). Therefore, controlling its production is of interest, especially in wine, in order to avoid alteration of the final product.

As described above, citrate metabolism is known to have a beneficial effect on both the growth and the acid stress resistance of LAB. However, Augagneur et al. (2007b) have reported a dual effect of citrate on the *O. oeni* strain ATCC BAA-1163. Indeed, they reported that while the addition of citrate in a culture medium had a beneficial effect on the growth of the bacterium at optimal pH, it completely impaired the growth at low pH. This observation is comforted by a recent study, in which an adaptive evolution to low pH has been performed on this strain and triggered the appearance of mutation in the *cit* locus. The mutations acquired by these evolved populations enabled them to growth under low pH in presence of citrate, while the ancestral strain was not able (Julliat et al., 2023). There is therefore no consensus on the effect of citrate on bacterial physiology, at least in *O. oeni*. In this review, citrate metabolism in LAB and its related citrate locus will be described with a focus on *O. oeni*. Subsequently, a genomic insight into citrate metabolism will be provided by describing the known regulatory mechanisms in different LAB. Finally, the beneficial effect of citrate on LAB will be described in more detail and the controversial effect on the acidic stress response in *O. oeni* referred to above will be discussed.

## 2 Citrate metabolism in LAB

Citrate metabolism in LAB includes three major steps, the first of which involves the uptake of citrate in its anionic or dianionic form through a specific permease called citrate permease. Citrate is then converted to acetate and oxaloacetate under the action of a citrate lyase and finally, oxaloacetate is degraded into pyruvate by an oxaloacetate decarboxylase. Pyruvate can then be reoriented to different pathways which will be more fully examined in the second part of this section. The enzymes described above are encoded by different genes organized in one or two citrate clusters depending on the LAB. Typical LAB citrate clusters are reported below.

### 2.1 Citrate cluster organizations

**Figure 1:**
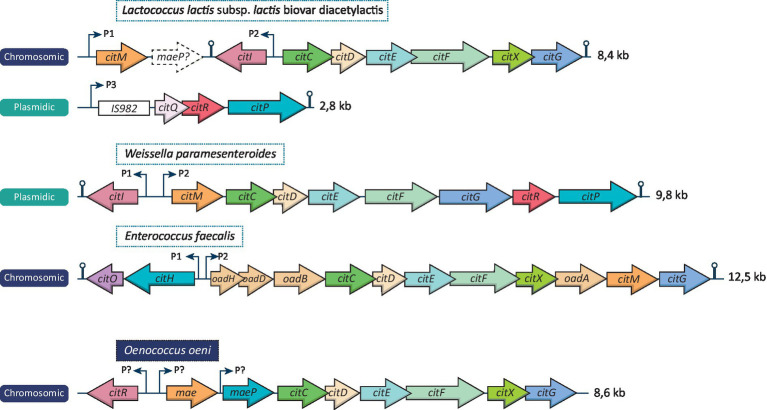
Organization of the citrate cluster from different LAB species. The citrate locus of *Lactococcus lactis* bv. diacetylactis, *Weissella paramesenteroides*, *Enterococcus faecalis* and *Oenococcus oeni* are represented and the size of each one is indicated. Thin arrows localize identified promotors (except for *O. oeni* in which putative promotors are represented). Air-pin structures represent identified rho-independent terminators. Dashed arrow represents a pseudogene founded on the citrate locus of *L lactis* bv. diacetylactis.

As mentioned above, not all LAB are able to metabolize citrate. Such metabolism implies the presence of genes encoding a citrate permease and a citrate lyase. These genes can either be localized in a plasmid or be integrated in the chromosome, depending on the LAB. Figure 1 represents different *cit* cluster organizations found in *Lactococcus lactis* subsp. *lactis* biovar diacetylactis, *Weissella paramesenteroides, Enterococcus faecalis*, and *O. oeni.* All these species shares common genes on their citrate locus: *citI,* which encodes a transcriptional activator of the operon belonging to the SorC/DeoR family (Martin et al., 2005), *mae/citM* encoding a soluble oxaloacetate decarboxylase enabling the conversion of oxaloacetate into pyruvate (Sender et al., 2004), *citP/maeP* encoding the citrate transporter (Ramos et al., 1994; García-Quintáns et al., 2008) and the *citC, D, E, F, X, G* genes which produce the different subunits of the citrate lyase and its activator components (Bekal et al., 1998a). This type of citrate cluster has been characterized in *L. lactis* bv. diacetylactis*, W. paramesenteroides, Leuconostoc mesenteroides* and *O. oeni* (Figure 1; Bekal-Si Ali et al., 1999; Martín et al., 2000; Mills et al., 2005). It should be noted that different names have been given to the citrate genes according to the LAB species, whereas in fact they share identical functions, a situation which may lead to confusion.

In other LAB, including *E. faecalis* and *Lacticaseibacillus casei,* certain variations in the *cit* cluster are noted. Indeed, the citrate cluster contains the gene *citO* encoding a transcriptional activator of the GntR family instead of *citI*, and the membrane-bound oxaloacetate decarboxylase is encoded by the *oadA,B,D* and *H* genes (Blancato et al., 2008; Mortera et al., 2013; Repizo et al., 2013). It will also be noted that certain LAB possess two genes encoding proteins with similar functions. This is the case for *L. lactis* bv. diacetylactis and *W. paramesenteroides* which possess two different genes encoding regulators: *citI* and *citR. E. faecalis* may also be cited in this regard as it possesses both the *citM* gene encoding the soluble oxaloacetate decarboxylase and the cluster *oadHDBA*, encoding the membrane-bound oxaloacetate decarboxylase complex. Furthermore, a pseudogene is found after *citM* on the *cit* locus of *L. lactis* bv. diacetylactis. Its sequence is similar to that of the *maeP* gene found in *O. oeni* but several frameshift mutations render it an unfunctional gene (Martín et al., 2004).

### 2.2 Citrate utilization pathways in LAB: catabolism of citrate into pyruvate

Citrate metabolism in LAB most commonly leads to the production of pyruvate, acetate and CO_2_. Three steps are necessary: first, citrate is taken up by the citrate permease encoded by the *citP, citH or maeP* gene depending on the LAB. In *L. lactis* bv. diacetylactis*, Lc. mesenteroides W. paramesenteroides* and *Lactiplantibacillus plantarum,* the *citP* gene encodes a transporter belonging to the 2-hydroxy-carboxylate transporter family (García-Quintáns et al., 2008; Yang et al., 2022). This antiporter catalyzes the exchange of the di-anionic form of citrate against lactate in most cases (Marty-Tesset et al., 1996; Bandell et al., 1998; Drider et al., 2004). In *La. casei*, citrate is taken up in complex with Ca^2+^ by a transporter belonging to the citrate-divalent metal ion family (CitMHS) encoded by the *citH* gene (Mortera et al., 2013). Finally, in *O. oeni*, the *maeP* gene encodes a uniport belonging to the Metabolite/H^+^ symporter family, enabling the uptake of citrate in its anionic form (Ramos et al., 1994). In this bacterium, another citrate transporter was suggested in the findings of Augagneur et al. (2007b) as its expression is positively regulated by the presence of citrate. This transporter is encoded by the *yaeP* gene but has not been characterized yet. Heterologous expression of this gene was carried out in order to characterize its function but was unsuccessful (Bonnin-Jusserand, 2011).

**Figure 2:**
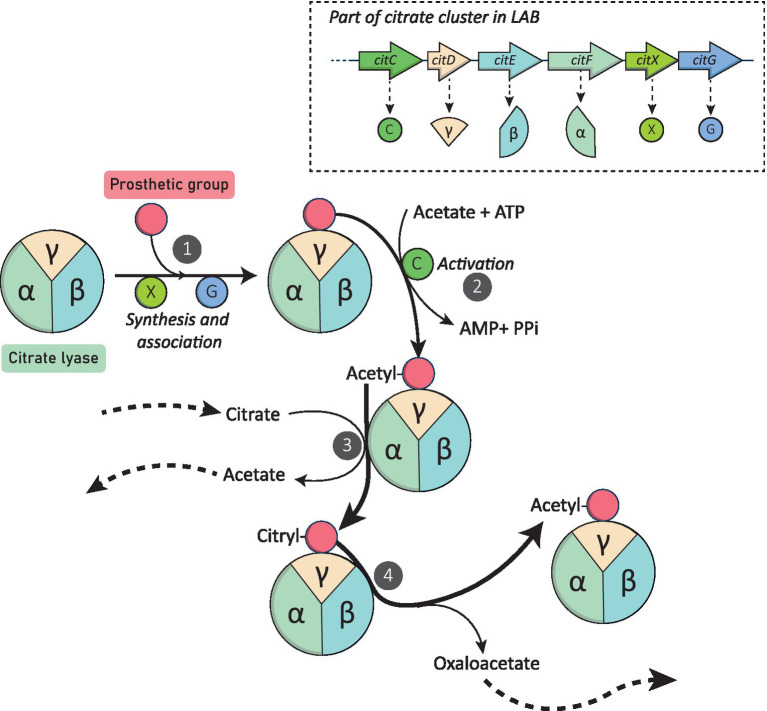
Citrate Lyase synthesis and catabolism of citrate mediated by this enzyme. Genes encoding the different citrate lyase (CL) subunits and the three additional proteins involved in this metabolism are showed. Arrows with solid lines represent enzymatic reactions, arrows with dashed lines represent the provenance or redirection of metabolites to other pathways. (1) CitX (apo-citrate lyase phosphoribosyl-dephospho-coenzyme A) and CitG (triphosphoribosyl-dephospho-coenzyme A synthase) synthetize the prosthetic group from ATP and dephospho-coenzyme A. (2) Activation by acetylation of the prosthetic group by CitC (acetate:SH-citrate lyase ligase). (3) Exchange of the acetyl group with the citryl group of citrate catalyzed by the α-subunit of the CL (acetyl-ACP:citrate ACP-transferase) and formation of acetate. (4) Clivage of the citryl group by the β-subunit of the CL (citryl-S-ACP oxaloacetate-lyase) releasing oxaloacetate and enable the acetyl group regeneration.

Citrate conversion into oxaloacetate and acetate is then initiated by the citrate lyase complex. This multimeric enzyme is composed by 3 subunits: γ (acyl carrier protein (ACP) containing the prosthetic group, EC: 4.1.3.6), α (acetyl-ACP:citrate ACP-transferase, EC:2.8.3.10) and β (citryl-S-ACP oxaloacetate-lyase, EC:4.1.3.34; 14, 55 and 34 kDa in *Lc. mesenteroides*, respectively; Bekal et al., 1998b). These subunits are encoded by the *citD, F* and *E* genes in LAB. Figure 2 illustrates the different steps required for the catabolism of citrate into acetate and oxaloacetate.

First, the α subunit of the citrate lyase catalyze the exchange of the citryl group (from citrate) with the acetyl group (linked to the prosthetic group of the ACP, the γ subunit of the citrate lyase) releasing acetate and citryl-S-ACP. Finally, the β subunit of the citrate lyase cleaves the citryl-S-ACP into oxaloacetate and acetyl-S-ACP, regenerating the acetyl group (Bekal et al., 1998a). This enzymatic activity is possible thanks to the action of three additional proteins: CitX (apo-citrate lyase phosphoribosyl-dephospho-coenzyme A, EC:2.7.7.61), CitG (triphosphoribosyl-dephospho-coenzyme A synthase, EC:2.4.2.52) and CitC (acetate:SH-citrate lyase ligase, EC:6.2.1.22), encoded by the *citX, citG*, and *citC* genes, respectively (Bott, 1997; Schneider et al., 2000). CitG and CitX participate in the formation of the prosthetic group from ATP and dephospho-coenzyme A, and CitC enables the activation of the latter via acetylation (García-Quintáns et al., 2008).

Acetate produced by citrate catabolism is released into the extracellular medium via passive diffusion or through a permease depending on the pHi of the bacteria, which can differ from one species to another. For example, in *L. lactis* bv. diacetylactis, it has been demonstrated that acetate can serve as the substrate of the citrate permease CitP and consequently be exchanged against citrate (Pudlik and Lolkema, 2011a). However, no exchange of citrate against one of its metabolic products by the permease MaeP could be demonstrated in *O. oeni* (Ramos et al., 1994). Another unidentified transporter of acetate presumably exists in this bacterium. While acetate is released, oxaloacetate, the second product of citrate, is metabolized into pyruvate and CO_2_ by an oxaloacetate decarboxylase (EC:1.1.1.38). The reaction leads to the consumption of one proton, generating a ΔpH which is one of the key steps in generating PMF (Lolkema et al., 1995). In many LAB, this reaction is catalyzed by a soluble oxaloacetate decarboxylase belonging to the malic enzyme family and encoded by the *citM* gene, also named *mae* depending on the LAB (Sender et al., 2004; García-Quintáns et al., 2008). In other LAB including *E. faecalis,* the reaction implies a membrane-bound oxaloacetate decarboxylase complex encoded by the *oadA, oadB, oadD*, and *oadH* genes (Repizo et al., 2013). Nevertheless, oxaloacetate is known to be unstable and can spontaneously decarboxylate into pyruvate by catalysis with a divalent metal ion (Sender et al., 2004; Pudlik and Lolkema, 2011b).

In the genus *Lactiplantibacillus*, oxaloacetate from citrate catabolism can also be metabolized into succinate using a part of the reductive Tricarboxylic Acid (TCA) cycle (Hugenholtz, 1993).

### 2.3 Fate of pyruvate

**Figure 3:**
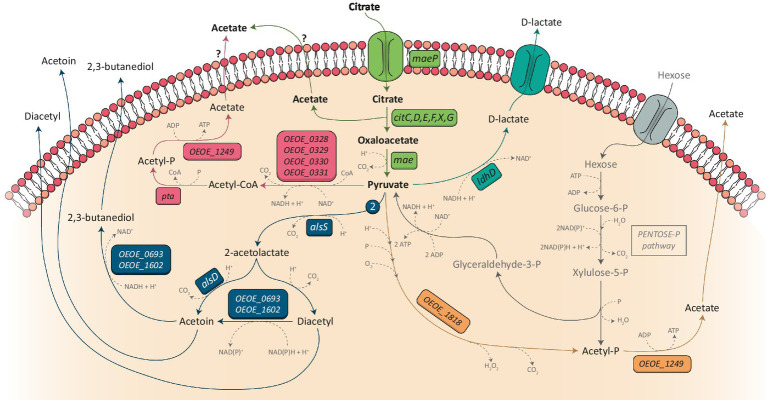
Citrate metabolism and becoming of pyruvate in *Oenococcus oeni*. Pyruvate produced by the citrate catabolism (green part) can be metabolized into D-lactate (blue pathway) for the reoxidation of co-factors generated by sugar metabolism (Pentose-P pathway, represented in gray). It can also be used for the production of acetate through the pyruvate dehydrogenase pathway (represented in pink) or through the pyruvate oxidase pathway (represented in orange). Finally, two molecules of pyruvate (symbolized by the number 2 on the figure) can be metabolized into α-acetolactate under the action of the acetolactate synthase (ALS) (dark blue pathway) which leads to the production of diacetyl, acetoin and 2,3-butanediol. Genes encoding the different enzymes are indicated in colored boxes. When no name was attributed to a gene, its locus tag from the PSU-1 strain is indicated. As the transport mechanism of acetate has not been characterized in *O. oeni*, question marks are represented.

Pyruvate is at the crossroads of several metabolic pathways known as the pyruvate node (Figure 3). Its metabolism can defer between heterofermentative and homofermentative LAB and the difference between the two has previously been reviewed (Gemelas et al., 2014). In the case of heterofermentative LAB such as *O. oeni* and *W. paramesenteroides,* pyruvate produced from citrate catabolism can be reoriented toward different pathways depending on the growth conditions (Contreras et al., 2018). In the presence of hexoses, it can be metabolized into D-lactate by a lactate dehydrogenase (EC:1.1.1.28) in order to reoxidize NADH coming from the phosphoketolase pathway (Zaunmüller et al., 2006). *In O. oeni*, the gene commonly linked to the lactate dehydrogenase is the *ldhD* gene (locus tag OEOE_0413 in PSU-1 genome; Kim et al., 2011; Yang et al., 2019). However, five other genes have been annotated as putative lactate dehydrogenases in this bacterium: OEOE_0025, OEOE_0701, OEOE_1182, OEOE_1672, and OEOE_1709. Nevertheless, only the products of the OEOE_0413 and OEOE_1672 genes have been detected in the proteome of the PSU-1 strain of *O. oeni* (Zaunmüller, 2008).

Pyruvate can also be used to produce ATP. In this way, pyruvate is decarboxylated into acetyl-CoA under the action of the pyruvate dehydrogenase, which strongly depends on the presence of coenzyme A and thiamin diphosphate (Hugenholtz, 1993). This reaction generates one molecule of CO_2_ and the reduction of one NAD^+^ into NADH + H^+^ which has to be reoxidize thanks to the transformation of another molecule of pyruvate into D-lactate by lactate dehydrogenase (Wagner et al., 2005; Zaunmüller et al., 2006). *Oenococcus oeni* possesses a pyruvate dehydrogenase which is annotated as the acetoin/pyruvate dehydrogenase complex (EC:1.2.4.1/2.3.1.12/1.8.1.4) encoded by the OEOE_0328, OEOE_0329, OEOE_0330, and OEOE_0331 genes. The acetyl-CoA produced is then metabolized into acetyl-P by a phosphotransacetylase (EC:2.3.1.8, encoded by OEOE_1435) which is then used by an acetate kinase (EC:2.7.2.1, encoded by OEOE_1249) to produce acetate and ATP (Wagner et al., 2005).

Another way to produce ATP from pyruvate without reducing co-factors is possible thanks to the action of the pyruvate oxidase (EC:1.2.3.3) encoded by OEOE_1818 in the *O. oeni* PSU-I strain. Indeed, this enzyme catalyze the conversion of pyruvate, inorganic phosphate and oxygen into acetyl-P, H_2_O_2_ and CO_2_, leading to the production of acetate and ATP from acetyl-P (Wagner et al., 2005). This reaction is only possible under aerobic conditions. It has recently been demonstrated that the channeling of pyruvate to acetyl-P production via the pyruvate oxidase pathway is greatly increased under acidic conditions (pH = 3) in *O. oeni* (Qi et al., 2021). However, this pathway must not be privileged in oenological conditions, as access to oxygen is limited.

Finally, pyruvate can be metabolized into C4 aroma compounds: diacetyl, acetoin and 2,3-butanediol. These C4 compounds are primarily obtained from pyruvate produced by citrate catabolism (McKay and Baldwin, 1990). When pyruvate accumulates in the cell, their synthesis increases in order to prevent a putative toxic effect of high pyruvate content. Indeed, it has been demonstrated that mutant strains of *Streptococcus lactis* or *L. lactis* bv. diacetylactis deficient in lactate dehydrogenase produced higher amount of α-acetolactate and diacetyl in the presence of citrate (McKay and Baldwin, 1974; Monnet et al., 2000). To complete this observation, Zuljan et al. have demonstrated that a *L. lactis* strain which was no longer able to metabolize pyruvate into C4 compounds became sensitive to the addition of this organic acid at low pH (Zuljan et al., 2014).

Two molecules of pyruvate and one thiamin diphosphate are needed to form α-acetolactate plus CO_2_ and the reaction is catalyzed by the acetolactate synthase (EC:2.2.1.6, encoded by the *alsS* gene in *O. oeni*; Hugenholtz, 1993; Garmyn et al., 1996; Olguín et al., 2009). The acetolactate synthase of *L. lactis* bv. diacetylactis showed a low affinity to pyruvate (Km = 50 mM), confirming that a high amount of pyruvate is needed to obtain C4 compounds (Snoep et al., 1992). Two pathways are then available to α-acetolactate: it can be metabolized into acetoin and CO_2_ by the α-acetolactate decarboxylase (EC:4.1.1.5), or it can be spontaneously oxidated into diacetyl and CO_2_ in the presence of oxygen. The α-acetolactate decarboxylase is encoded by the *alsD* gene in *O. oeni* and is organized in a single operon with the *alsS* gene (Garmyn et al., 1996). Moreover, the expression of this gene could be induced by the presence of ethanol (Olguín et al., 2009). Diacetyl is the major molecule which imparts a buttery aroma to wine, and it is primarily synthetized from pyruvate derived from citrate metabolism. Nevertheless, it has been proven than other metabolisms can contributes to its production, including sugar and malate metabolism (Mink et al., 2014, 2015; Sternes et al., 2017) The highest amount of diacetyl is detected during malolactic fermentation when almost all malic acid and more than half the citrate concentration are consumed (Mink et al., 2014). this amount must not exceed 1 to 4 mg/L. At a concentration more than 7 mg/L, diacetyl will impart an undesirable buttery flavor to wine (Bartowsky and Henschke, 2004). Thus, controlling its production is of interest. Unfortunately, the regulation of citrate metabolism in *O. oeni* is to date imperfectly understood. This reflection will be the subject of a paragraph bellow in which the regulation of citrate metabolism in other LAB will be described, enabling a parallel with *O. oeni* to be drawn.

Finally, diacetyl can be reduced to acetoin which itself can be reduced to 2,3-butanediol. The reaction requires NADH or NADPH as a co-factor, depending on the LAB (Hugenholtz, 1993). According to a study on *L. lactis* bv. diacetylactis, these two reactions could be metabolized by the same enzyme, namely acetoin reductase (EC:1.1.1.304/1.1.1.47), and this enzyme may have a greater affinity for diacetyl (Crow, 1990). In the *O. oeni* PSU-1 genome, two genes are annotated as acetoin reductase: OEOE_0693 and OEOE_1602. According to previous studies (Cogan, 1981; Hugenholtz, 1993), this enzyme may be partially inhibited in presence of citrate.

## 3 A genomic insight into citrate metabolism: way of regulation pathway

The extreme diversity of the citrate locus in LAB was mentioned above. Indeed, the gene organization and the localization of the operon but also the genes within the locus are not the same depending on the species. In light of this diversity, regulation of citrate locus expression could be species-dependent; this may also be true because the growth environment of each LAB is very different, notably in terms of pH conditions. It has indeed been demonstrated that environment can impact the expression of the citrate locus in different ways depending on the species. In this section, the mechanisms of citrate locus regulation described in the literature for certain LAB will be detailed. The effect of environmental parameters on the expression of the operon will then be discussed.

### 3.1 Regulation of citrate locus expression

Previous studies have explored the regulatory mechanism of citrate locus expression in various LAB. Findings highlight an activator protein named CitI, CitR, or CitO acting at the transcriptional and/or the post-transcriptional level.

In *W. paramesenteroides*, the genes related to citrate metabolism are organized in a single plasmid-located cluster of 9.8 kb. Two divergent promoters located between *citI* and *citM* have been identified: the first (P*citI*) controls *citI* expression and the second (P*cit*) ensures the transcription of the whole *citMCDEFGRP* operon (Martín et al., 2000). In addition to the *citM, citP* and *citCDEFG* genes, which encode the key proteins involved in the citrate pathway, i.e., oxaloacetate decarboxylase, citrate permease and the different subunits of citrate lyase, respectively, this cluster includes two genes encoding putative regulators: *citI* and *citR.* CitI is a protein belonging to the SorC family. Its role in the expression of the *citMCDEFGRP* cluster has been explored (Martín et al., 2000; Martin et al., 2005). Indeed, the role of CitI as a transcriptional activator in *citMCDEFGRP* cluster expression has been revealed via heterologous expression in *E. coli*. The intergenic region citI-citM is rich in A/T bases (77%) with an intrinsic bending. Two operator sites recognized by CitI (O1 and O2) have been identified on this region and CitI is able to bind cooperatively to them. The affinity of CitI for its DNA operators is directly enhanced by citrate, resulting in increased RNA polymerase recruitment at Pcit and PcitI. In this way, in the absence of citrate, CitI interacts weakly with operator sites, resulting in a low level of expression of the citrate pathway enzymes such as the citrate permease which ensures the uptake of citrate inside the cell. When citrate is available in the environment, it is transported inside the cell and directly increases the affinity of CitI for its DNA operator, leading to an increase in the *cit* locus expression (Martin et al., 2005). It appears that similar operator sites can also be found on the citrate locus promoter regions of other LAB, including *O. oeni.* Thus, Martin et al. (2005) suggested that this regulatory mechanism may be similar in other LAB. Concerning the *citR* gene of this bacterium, the role of its product on *cit* cluster expression has not yet been investigated.

In addition, the *citMCDEFGRP* cluster expression in this bacterium is subjected to post-transcriptional regulation by a specific processing at the level of complex structures probably recognized by endonucleases (Martín et al., 2000). Thus, three different transcripts of the *cit* cluster have been detected: an 8.8 kb RNA including the transcript of all the *citMCDEFGRP* genes, a 6.1 kb RNA including the transcript from *citD* to *citP* and finally a 7.2 kb RNA including the transcript from *citM* to *citR* (Martín et al., 2000). The authors have suggested that this RNA processing might enable regulation of the synthesis of the different proteins in suitable proportions (Martín et al., 2000).

In *L. lactis* bv. diacetylactis, the genes encoding proteins involved in citrate metabolism are organized in two different clusters: the *citQRP* operon which is located on a plasmid and the *citMCDEFXG* operon which is integrated in the chromosome (Martín et al., 2004). This second cluster also includes the *citI* gene which is oriented in divergent direction. Promoter regions have been identified: before the IS element for *citQRP* cluster expression, before *citM* for *citMCDEFXG* expression and another one in a divergent direction before *citI* (de Felipe et al., 1994; Martín et al., 2004). The promoter enabling *citQRP* expression and the one before *citM* are very similar, rich in A/T bases (Martín et al., 2004). The two citrate clusters include two genes encoding regulatory proteins: *citI* and *citR.* The impact of CitR on permease gene expression has been explored by heterologous expression (de Felipe et al., 1994). This protein of 13.1 kDa is encoded by the 339 bp *citR* gene and shares homology with the *citR* of *W. paramesenteroides*. Overexpression of *citR* had no impact on the transcription of *citP*, whereas reporter gene activity decreased significantly. Thus, CitR could act as a repressor at a post-transcriptional level. Another experiment was conducted in which the *citR and citP* genes were both fused with the reporter gene; the construction was under the control of a constitutive promotor. This experiment revealed that the introduction of a frameshift mutation on *citR* led to decreased activity of the reporter gene. This finding implies that the coupled translation of *citR* and *citP* enhances the expression of *citP*. Thus, CitR in *L. lactis* bv. diacetylactis acts at a post-transcriptional level and may have two different impacts on the expression of *citP*. A secondary structure of the mRNA was detected before *citR* including its ribosome-binding site and the start codon. One of the hypotheses is that CitR could help to stabilize this secondary structure, preventing the coupled translation of *citR* and *citP*. The impact of CitR on the second citrate cluster of *L. lactis* bv. diacetylactis has not yet been explored.

The *citMCDEFXG* of *L. lactis* bv. diacetylactis includes the *citI* gene between *citM* and *citC* in reverse orientation. This 930 bp gene encodes a 34.8 kDa regulatory protein named CitI. Its effect on the expression of the two *cit* clusters of *L. lactis* bv. diacetylactis has not yet been investigated. Nevertheless, Martín et al. (2004) have suggested that a post-transcriptional regulation of the *citI* expression is possible via an RNA-silencing mechanism, as both sense and antisense transcripts of this genomic location were detected by RT-PCR. Indeed, this gene can be transcribed from the promoter before *citM* (P1) and from its own promoter (P2; Figure 1). Furthermore, it has been demonstrated that citrate lyase activity increases at pH 5 compared to pH 7 and that more *citI* transcripts are detected in the acidic condition (Martín et al., 2004). Thus, a regulatory mechanism similar to that identified in *W. paramesenteroides* was proposed by the authors: CitI would seem to be a transcriptional activator of the *citMCDEFXG* cluster, whose expression would presumably be enhanced by a decrease in pH.

In the case of *E. faecalis*, there is another regulatory protein named CitO belonging to the GntR family and clustered in the FadR C-terminal Domain family. It has been shown to act as a transcriptional activator of the *cit* cluster expression in this bacterium, as no citrate metabolism could be detected when the *citO* gene was mutated (Blancato et al., 2008). Molecular modifications induced by citrate and metal ions (Ni^2+^, Zn^2+^) in the C-terminal domain of CitO are required for optimal transcriptional activation by CitO (Blancato et al., 2008). The authors have also identified two operator sites on the promoter region of *citO*, and citrate seems to interact with CitO, modulating its capacity to bind DNA. Similar to what has been suggested in the cases of *W. paramesenteroides* and *L. lactis* bv. diacetylactis, the *cit* cluster of this LAB could be the subject of post-transcriptional regulation, as four secondary structures have been identified on the operon and as smaller RNA were also detected (Blancato et al., 2008).

In *O. oeni*, no study has been undertaken to understand the role of CitR, the putative regulator included in its citrate operon. Compared to all the regulator proteins mentioned above, the CitR protein of *O. oeni* seems to be closest to the CitI protein of *L. lactis* bv. diacetylactis, as they share 52% of their amino acid identity. As previously mentioned, two putative operator sites for this protein have been proposed by Martin et al. (2005), presumably located between *citR* and *mae*. Concerning promoting regions, two putative promoters were identified with sequences which could be related to the −10 and −35 regions: the first one seemingly before *mae* and, similarly to other LAB, it could enable the entire operon transcription. This promoter is also rich in A/T bases, as described in *W. paramesenteroides* and in *L. lactis* bv. diacetylactis. Typical regulatory regions −35 and −10 were also found before *maeP*, meaning that the citrate operon could also be expressed from this putative promoter in this bacterium. Another promoter must be located before the *citR* gene, as it is divergently expressed. Nevertheless, identifying the −35 and −10 regions near this gene is not evident, nor is the ribosome binding site. Determining the +1 transcription start site would be necessary to precisely define the promoting area of *citR*. Similarly to what was found in other LAB, the *cit* cluster expression of *O. oeni* could be regulated post-transcriptionally.

### 3.2 Effect of environment on the citrate locus expression

As mentioned above, the environment can affect the expression of the citrate cluster in LAB. Indeed, in most LAB, citrate induces the expression of the operon. This positive effect of citrate has been reported in *W. paramesenteroides, Lc. mesenteroides, Limosilactobacillus panis*, and *E. faecalis* (Bekal-Si Ali et al., 1999; Martín et al., 2000; Blancato et al., 2008; Kang et al., 2013). On the contrary, in *L. lactis* bv. diacetylactis, the expression of the *cit* cluster is induced at low pH while citrate did not enhance the *cit* transcript level detected (Martín et al., 2004).

In *O. oeni*, a previous RT-qPCR study on the ATCC BAA-1163 strain has demonstrated that neither citrate nor pH had an effect on the expression of *mae, maeP*, or *citF* (Augagneur et al., 2007b). These results are not in accordance with transcriptomic analysis performed on RNA of the SD-2a strain, which demonstrated an up-regulation of the citrate lyase genes and *mae* at low pH (Liu et al., 2017). Regarding the sequence of the citrate operon in the ATCC BAA-1163 strain, one base deletion can be noted in the sequence of the *citR* gene compared to other strains, leading to the appearance of a premature stop codon (length of 256 amino acids instead of 320). Thus, the putative regulator CitR might be unfunctional in this strain and the conclusions drawn from the data collected on this strain would therefore not be generalizable to the whole species. Indeed, studies on *W. paramesenteroides* have shown the direct positive effect of citrate on the expression of the regulator *citI* specifically (Martín et al., 2000). If the regulator is unfunctional in the ATCC BAA-1163 strain of *O. oeni,* this might explain why no variation of the *cit* locus expression could be noted in the experiment conducted by Augagneur et al. (2007b), despite the addition of citrate and the acidic conditions. More experiments based on the characterization of different *O. oeni* strains would be necessary to confirm this hypothesis and to identify which environmental parameters affect the *cit* locus expression in this bacterium. Other RTqPCR and transcriptomic analyses on *O. oeni* grown in “wine-like” conditions have shown a significative induction of certain genes of the citrate locus by wine-related stresses, especially ethanol (Olguín et al., 2009, 2010; Bordas et al., 2015; Margalef-Català et al., 2016). Moreover, another study has demonstrated an over-expression of *citE* induced at low pH but also by the presence of fructose (Pretorius et al., 2019). The presence of phenolic compounds also seems to have an impact on *O. oeni* citrate metabolism and acetate production (Rozès et al., 2003; Campos et al., 2009). It would be of interest to characterize in further detail the impact of all these environmental parameters on the *cit* locus expression of *O. oeni*, especially on the expression of *citR* encoding the putative regulator. A deeper understanding of citrate metabolism regulation in *O. oeni* would help to better grasp one of the stress response mechanisms of this bacterium, as it has been reported in several instances that these genes are up-regulated during adaptation to wine (Bordas et al., 2015; Margalef-Català et al., 2016).

Concerning diacetyl, its production by *O. oeni* is influenced by the presence of oxygen, the redox potential of the wine and the concentration of pyruvate and citrate (Nielsen and Richelieu, 1998; Mink et al., 2015). Therefore, understanding the circumstances of citrate metabolism activation could allow for better control of the citrate-associated synthesis of products like diacetyl, which may impact positively but also negatively the aromatic profile of wines.

## 4 Beneficial effect of citrate on LAB growth and stress resistance

The citrate pathway is an unstable trait in LAB, as some of the key genes are plasmid-located in several species (Hugenholtz, 1993). Furthermore, it has been reported for some LAB that citrate itself does not support the growth of bacteria (García-Quintáns et al., 2008). However, citrate confers some interesting advantages on bacteria which are able to metabolize it. Indeed, this metabolism generates PMF and leads to the consumption of several scalar protons, helping to maintain pHi in an acidic environment (Poolman et al., 1991; Ramos et al., 1994). Furthermore, in heterofermentative LAB, pyruvate produced from citrate can be further metabolized into D-lactic acid to reoxidize cofactors resulting from sugar metabolism. Thus, cometabolism of carbohydrates and citrate can significantly enhance the growth of bacteria (Salou et al., 1994; Ramos and Santos, 1996; Kang et al., 2013).

### 4.1 Proton motive force generation

PMF is one of the main mechanisms by which energy is generated in LAB. PMF consists of a chemical (ΔpH) and an electrical gradient (ΔΨ), also called membrane potential. Two systems can generate PMF: the primary transport system, which consists of the translocation of one proton out of the cells and, in this case, the generation of chemical and electrical gradient is coupled, and the secondary transport system. Citrate metabolism belongs to the second category. In this case, pH gradient and membrane potential occur at two different steps of the metabolism. Indeed, membrane potential is generated by the transport of citrate into the cells while ΔpH occurs when citrate is metabolized (Ramos et al., 1994). The free energy released by PMF is strong enough to activate the F_0_-F_1_ ATPase to produce ATP (Poolman et al., 1991; Poolman, 1993; Ramos et al., 1994). To better describe this phenomenon, the example of *L. lactis* bv. diacetylactis will be taken and compared to that of *O. oeni*.

**Figure 4:**
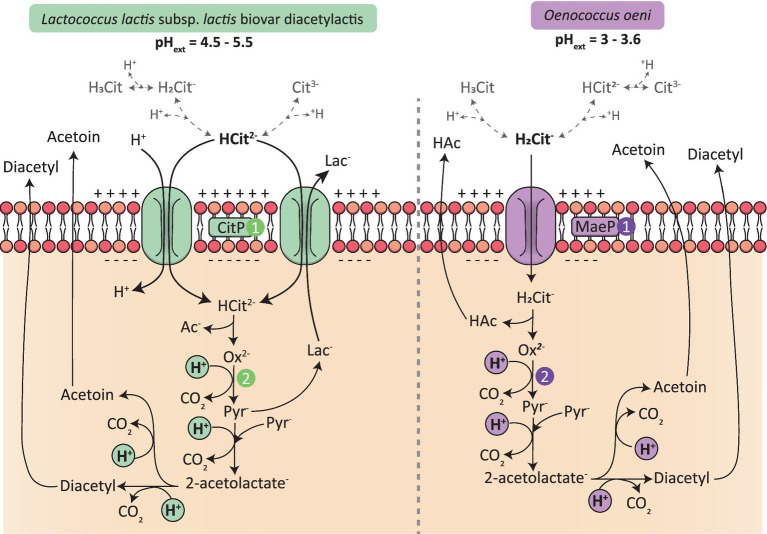
Generation of PMF in *Lactococcus lactis* bv. diacetylactis and in *Oenococcus oeni* by citrate metabolism. 1/ Internalization of citrate by either the permeases CitP or MaeP leads to the creation of an electrical gradient, inside negative (ΔΨ). Citrate forms are represented in gray, the majority form founded in each environment of the two LAB according to the extracellular pH is in bold 2/ Citrate metabolism drives the consumption of scalar protons leading to the creation of a ΔpH gradient, inside alkaline.

*L. lactis* bv. diacetylactis is used for milk fermentation and converts lactose to lactate, resulting in the acidification of the medium, from a pH of 7 to a pH of around 4 (García-Quintáns et al., 1998). It has been proven that citrate metabolism is induced at low pH in this bacterium, where the highest uptake rates were observed at pH 4.5 to 5.5 (Magni et al., 1994; Martín et al., 2004). The pKa of citrate are 3.13, 4.76 and 6.40 (Martell and Smith, 1982). Thus, when the pH of the medium is between 4.5 and 5.5, citrate will be principally found in the Hcitrate^2−^ form. This form of citrate is the one that is taken up by the permease CitP (Figure 4; Magni et al., 1996). This transporter can act as a symporter, meaning that citrate is co-transported with one proton (Magni et al., 1996). It might also act as an antiporter, as has been described for *Lc. mesenteroides* and *L. lactis*; in this case, citrate is exchanged with one molecule of lactate (Figure 4; Marty-Tesset et al., 1996; Bandell et al., 1998; Pudlik and Lolkema, 2011a). In each case, the transport of citrate leads to the translocation of one negative charge into the cell, creating a negative electrical gradient inside (Magni et al., 1994; Marty-Tesset et al., 1996). Then, the decarboxylation of oxaloacetate into pyruvate leads to the consumption of a scalar proton and creates the second aspect of PMF: the pH gradient (Lolkema et al., 1995).

*Oenococcus oeni* grows in more acidic environments than *L. lactis* bv. diacetylactis, with the pH of wine reaching between 3 and 3.6. Thus, in wine, citrate is mainly found in the anionic form H_2_citrate^−^. This form of citrate is the particle transported by the permease CitP (Ramos et al., 1994). The latter acts as a uniporter leading to the internalization of one negative charge (ΔΨ) and it has been proven that citrate cannot be exchanged by one of the products of citrate metabolism, unlike what has been demonstrated in other LAB (Ramos et al., 1994; Marty-Tesset et al., 1996; Bandell et al., 1998). The second part of PMF is created by the consumption of scalar protons, which occur during the catabolism of citrate (Figure 4; Ramos et al., 1994). The generation of PMF is of importance as it is one of the only ways for *O. oeni* to produce energy in wine, because of sugar starvation. Thus, in the same way as malolactic fermentation, which represents the major source of PMF generation in *O. oeni*, citrate metabolism participates in the survival of the bacterium in a harsh environment such as wine.

### 4.2 Intracellular pH homeostasis

LAB grow in acidic environments, in particular *O. oeni* which has to survive in wine that can sometimes reach pH lower than 3. At low pH, many acids are found in their protonated form, meaning that they can enter the cells by simple passive diffusion. The consequence is a lowering of pHi, which can trigger the denaturation of proteins, decreasing enzyme activity and leading to the rigidification of the membrane, affecting transporter activity (Tourdot-Maréchal et al., 2000; Grandvalet et al., 2008). All of these changes modify cell homeostasis and can seriously affect the survival of bacteria. Extruding protons by the F_0_ F_1_-ATPase is one of the mechanisms used by LAB to maintain their pHi, but as it is energy-consuming it needs to be a temporary response. As mentioned above, citrate metabolism leads to the consumption of several protons and its catabolism in weaker acids such as lactate or acetate contributes to the maintain of pHi. It has also been reported that citrate metabolism relieved growth inhibition caused by an accumulation of lactate produced by glycolysis in the cytoplasm of *L. lactis,* thanks to the exchange of citrate against lactate by the permease CitP (Magni et al., 1999). Moreover, another study on *L. lactis* has demonstrated the beneficial effect of C4 compound synthesis against pyruvate accumulation which can become toxic at low pH. Indeed, the disruption of the *als* gene encoding the acetolactate synthase led to the loss of cell viability when grown at low pH in the presence of pyruvate, due to the cells’ inability to regulate their pHi (Zuljan et al., 2014). In the same way, disruption of the *mae* gene in *L. lactis* leads to an intracellular oxaloacetate accumulation which significantly impacts growth, especially at low pH (Augagneur et al., 2007a; Pudlik and Lolkema, 2011b). There is no doubt that citrate metabolism is one of the stress response mechanisms used by LAB to resist in acidic environments.

### 4.3 Co-metabolism of citrate and glucose

LAB are able to ferment sugars from their environment to produce energy via two different pathways: homofermentative metabolism which corresponds to glycolysis (also called the Embden-Meyerhof-Parnas pathway) and which mainly leads to the production of lactic acid, and heterofermentative metabolism which uses the phosphoketolase pathway and produces lactic acid, but also ethanol, acetic acid and CO_2_ (Kandler, 1983). Heterofermentative LAB can also be divided into two different groups: the facultatively heterofermentative, which are able to ferment sugars through glycolysis and the phosphoketolase pathway, and the obligately heterofermentative, such as *O. oeni*, which lack the main enzyme involved in the glycolysis pathway, resulting in the incapacity to use this metabolism (Salvetti et al., 2013).

The phosphoketolase pathway leads to the production of 1 or 2 molecules of ATP and is thus an energy source for LAB. Fructose and glucose, which are the most common hexoses founded in LAB environments, are metabolized to glyceraldehyde-3-P, acetyl-P and CO_2_ and this reaction requires the reduction of two NAD(P)^+^ (Figure 3). Glyceraldehyde-3-P is then used for the production of pyruvate which leads to the reduction of one extra NAD^+^, although this one is quickly reoxidized under the action of the lactate dehydrogenase which produces lactate from pyruvate (Unden and Zaunmüller, 2009). The two first NAD(P)H produced by hexose fermentation also need to be reoxidized. This reoxidation is carried out by the conversion of acetyl-P to ethanol, but it appears that this pathway is very slow in *O. oeni* compared to the high activities of the enzymes of the phosphoketolase pathway (Maicas et al., 2002; Unden and Zaunmüller, 2009). Other pathways linked to sugar metabolism are used by the bacterium to reoxidize these cofactors: the erythritol, glycerol, and mannitol pathways (Maicas et al., 2002; Richter et al., 2003; Zaunmüller et al., 2006). Nevertheless, external electron acceptors may also be employed, as in the case of citrate metabolism. Indeed, citrate metabolism produces pyruvate, which can be converted by lactate dehydrogenase to D-lactate, thus enabling the reoxidation of one cofactor (Ramos and Santos, 1996; Zaunmüller et al., 2006). Consequently, it has been demonstrated that the growth of *O. oeni* is enhanced in medium containing both citrate and glucose, compared to a condition with only glucose (Salou et al., 1994; Ramos and Santos, 1996). A similar conclusion was drawn in a study on *Li. panis,* in which the addition of citrate caused a metabolic shift as more acetate was produced from glucose than ethanol, resulting in higher ATP production (Kang et al., 2013).

On the other hand, in homofermentative LAB, the association between citrate and sugar metabolisms appears only when citrate is exchanged by the end-product of glycolysis, i.e., lactate. In this case, it is the sugar metabolism which has a beneficial effect on citrate metabolism, as the addition of glucose enhances citrate uptake by bacteria (Pudlik and Lolkema, 2011a). Nevertheless, the co-metabolism of citrate and glucose also results in growth advantage in homofermentative LAB (Starrenburg and Hugenholtz, 1991; Sánchez et al., 2008).

## 5 A controversial effect of citrate metabolism highlighted in *Oenococcus oeni*

Several beneficial effects of citrate metabolism on LAB growth have been described above. However, a dual effect of citrate on *O. oeni* acid stress resistance has been highlighted (Augagneur et al., 2007b; Julliat et al., 2023). Indeed, while the addition of citrate in FT80m medium resulted in a higher final biomass and growth rate achieved at pH 5.3, the optimal pH for the growth of *O. oeni*, a significant inhibition effect was observed on bacterial growth in a pH 3.2 medium containing citrate (Augagneur et al., 2007b). In addition, an experimental evolution performed to improve *O. oeni* acid tolerance led to the appearance of fixed mutations in the *cit* locus. These mutations triggered down-regulation of citrate genes resulting in a slowdown in the rate of citrate consumption in the evolved populations tested. This study demonstrated that while the growth of the ancestral strain is impaired by the addition of citrate in pH 3 FT80m, that of evolved populations is not affected (Julliat et al., 2023). It demonstrated a correlation between citrate consumption rate and acid tolerance in *O. oeni*. The growth inhibition observed may be due to an inability of the bacterium to maintain its pHi. According to Augagneur et al. (2007b), citrate itself did not seem to directly affect the pHi maintenance. Consequently, the authors turned their attention to one of the products of citrate metabolism: acetate. This weak acid (pKa = 4.74) is primarily found in its protonated form at pH lower than 4.8 and can therefore easily re-enter the cells when the pH of the medium is low enough. Experiments conducted by Augagneur et al. (2007b) have shown a significant inhibitory effect of acetate addition on the growth of bacteria and, to a lesser extent, on the maintenance of pH gradient and membrane potential. Thus, the quick production of acetate from citrate metabolism but also sugar metabolism could become toxic for the cells. In addition, pyruvate production could also play a role in this case, as it has been shown in *L. lactis* that an accumulation of pyruvate that could no longer be metabolized into C4 compounds as a result of *alsS* mutation led to the loss of cell viability (Zuljan et al., 2014).

These studies may bring to light a controversial effect of citrate at low pH specific to the bacterium *O. oeni*. Nevertheless, it must be kept in mind the experiments conducted by Augagneur et al. (2007b) were performed on a laboratory complemented by 20 mM of citrate, corresponding to 3.8 g/L. This concentration is around 10-fold higher than what can be found in wine, meaning that the testing conditions were well removed from the real environment of the bacterium (Alexandre et al., 2008). Furthermore, the experiments were conducted using the laboratory strain ATCC BAA-1163. As noted above, this strain possesses a one-base deletion on the *citR* sequence compared to other strains, resulting in the appearance of a premature stop codon. It is thus possible that ATCC BAA-1163 has a particular behavior regarding citrate consumption.

No additional study has been carried out on *O. oeni* to demonstrate the effect on the bacterium of citrate addition at low pH. Nevertheless, other experiments have been conducted on other strains of *O. oeni* following the consumption of sugars and organic acids at low pH in presence of citrate. In these studies, the bacteria consumed all malic acid, whatever the pH (between 3 and 3.5 in some conditions tested) or the citrate concentration (0.5 g/L in the study of Pretorius et al., 2019 and between 5 to 8.5 g/L in the study of Viljakainen and Laakso, 2000). This was not the case in the study of Augagneur et al. (2007b) as the addition of citrate at pH 3.2 prevented the total malate consumption. This suggests that bacteria were not affected by the presence of citrate at low pH in the conditions tested by Viljakainen and Laakso (2000) and Pretorius et al. (2019). Other studies would be needed to generalize the controversial effect of citrate on the acid resistance of *O. oeni* observed by Augagneur et al. (2007b) and to determine whether this metabolism could have an impact on malolactic fermentation.

## 6 Conclusion and perspectives

This review aimed to summarize current understanding of citrate metabolism in LAB with a focus on the wine bacterium *O. oeni.* This metabolism requires 3 different complex proteins: a permease, a citrate lyase and finally an oxaloacetate decarboxylase (Hugenholtz, 1993; Bekal et al., 1998a; Drider et al., 2004). The genes encoding these enzymes are organized in a long citrate operon which can be included in a plasmid or integrated in the bacterial chromosome, depending on the LAB (de Felipe et al., 1994; Drider et al., 2004; Martin et al., 2005; Blancato et al., 2008). The regulation mechanism of the citrate locus expression has been described in some LAB, bringing to light the major role of a transcriptional/post-transcriptional activator encoded by a gene named *citI, citR*, or *citO,* depending on the LAB. We can note in passing that the different names assigned to the various regulatory proteins reveal a certain lack of relevance, as the *citR* encoding the regulatory protein found in *O. oeni* is genetically closer to *citI* than to the *citR* of *L. lactis* bv. diacetylactis.

Expression of the citrate locus may be induced by environmental parameters such as the presence of citrate or pH (Bekal-Si Ali et al., 1999; Martín et al., 2000, 2004; Blancato et al., 2008). In *O. oeni*, certain RT-qPCR and RNAseq studies have demonstrated the up-regulation of some citrate locus genes by wine-related stresses such as ethanol (Bordas et al., 2015; Margalef-Català et al., 2016), but further experimentation would be necessary to decrypt the regulation mechanism of the citrate operon in this specific species.

Citrate metabolism has a beneficial effect on growth and acid stress resistance in LAB. Nevertheless, a controversial effect of citrate has been highlighted on the acid stress resistance of the ATCC BAA-1163 strain of *O. oeni* (Augagneur et al., 2007b; Julliat et al., 2023). This effect could be strain-dependent, as no other study reported this kind of effect of citrate at low pH on this bacterium. It would be of interest to study citrate metabolism in different *O. oeni* strains to confirm this hypothesis. In addition, a complementary study could be undertaken in order to understand how citrate may be the object of growth inhibition in this strain. Augagneur et al. (2007b) suspected acetate production to be toxic, but pyruvate could also play an important role, as has been demonstrated in *L. lactis* (Zuljan et al., 2014). From a more general point of view, further knowledge is needed on the citrate metabolism of *O. oeni* and particularly on how this metabolism is regulated in the species. No study to date has been undertaken to describe the role of CitR, the putative regulator of the citrate operon in *O. oeni*, and little information is available concerning the effect of environment on the expression of the *cit* cluster. As far as previous studies have allowed us to ascertain, citrate metabolism does not seem to impact malolactic fermentation (Viljakainen and Laakso, 2000; Pretorius et al., 2019). However, to the extent that this metabolism may be strain-dependent, conducting fermentation with different *O. oeni* strains at different pH and with different citrate concentrations following malate and citrate consumption could be a profitable way forward. Finally, as citrate metabolism leads to the production of diacetyl, a C4 compound responsible for the buttery aroma that is only desirable in small amounts in wine (Bartowsky and Henschke, 2004), a more precise understanding of how environmental conditions affect citrate metabolism could enable a better control of diacetyl production.

## Author contributions

CE: Writing – review & editing. JC: Writing – review & editing, Funding acquisition. MF: Writing – review & editing, Funding acquisition. HA: Writing – review & editing, Supervision. CR: Writing – review & editing, Supervision. CG: Writing – review & editing, Supervision.
